# Event and Comment

**Published:** 1913-07-15

**Authors:** 


### DENTAL REGISTER.

**Vol. LXVII. July 15, 1913. No. 7**

### EVENT AND COMMENT.

THE DENTAL RESEARCH PROBLEM. Considerable activity seems just now manifested in interesting the profession in some form of organized and professionally supported scientific research. The committee of the National Association, headed by Dr. Price, seems to be making quite satisfactory progress in raising funds and providing suitable places for research. Another effort is being fostered by the Delta Sigma Delta fraternity to raise, by a per capita tax, sufficient funds to keep some fraternity brother at this work. Several of the endowed scientific organizations have also expressed special interest and have offered to extend such aid as they could to any promising scheme for definite results. Heretofore our scientific work has been the result of individual effort and sacrifice by loyal and devoted members of the profession to a considerable extent. It would seem that we are now sufficiently conscious of the need and value of this kind of effort to engage in it with more professional co-operation than ever before, and the spirit of helpfulness is so apparent that some satisfactory outcome of the present aggitation is assured. We are not wise enough to venture a plan of action, and we very much doubt if any one will be able to conceive one that will harmonize all our ideals, but we should, nevertheless, do the best our wisdom and opportunities will permit. In our enthusiasm we should not undertake so large a task as to invite possible failure, and yet we must undertake a worthy objective. It would seem that the most conservative plan would be to utilize as many as possible of the present going concerns as could be manned at reasonable cost, with dental research men of some experience, rather than to spend large sums on new equipment and untried men. Scientific research is disappointing at best, because a long and tedious experiment more often fails to produce satisfactory results than to reach a desired conclusion. If such a result happens to an experiment financed by the profession, there is bound to be impatience and a falling off in the financial support. An enterprise of that kind also puts the worker at a disadvantage as he is expected to make good; whereas, an experienced and established worker has an accumulated reserve with which he can satisfy reasonable expectations and may be expected to make some definite progress.

PREVENTION OF DENTAL CARIES. We are reprinting in this issue two papers, in abstracted form, published in full in the *Journal of Allied Societies*, on the general topic of prevention, which on first reading seem so contradictory as to lead to the conclusion that we are not in accord as to a practicable method of preventing caries of the teeth. The first article ignores, to some extent, the traditional practice of keeping the teeth free from extraneous deposits of various kinds by means of the tooth brush and chemical detergents; while the other censures the profession for not promulgating a campaign of educating the people as to the proper mouth toilet and the use of safe and wholesome detergent means and methods. The former is contributed by a dentist, Dr. H. A. Kelley, of Portland, Me., and the latter by a school teacher, Prof. H. C. Kelly, of Springfield, Mass., to the Massachusetts Dental Society. Their viewpoints, of course, differ as well as their visions, and each presents an excellent statement of our present knowledge with a conclusion that is fully justified. A careful review of both papers leads to the conclusion that there may be a large measure of truth in each, and that the situation is not so paradoxical as it seems. There can be little doubt but the campaign for oral cleanliness by the tooth toilet method has been overdone to the extent that it has caused people to rely wholly on these artificial methods of freeing the mouth from fermentable debris of various sorts. We predict, on the contrary, that we shall make an equally disastrous mistake by any attempt, however wisely made or religiously executed, which is based wholly on the theory that oral sanitation can be assured by careful study and adoption of an aseptic dietary. Many years ago, Dr. George Watt advocated the liberal use of free acids, as in vinegar, lemons and fruits for patients with the so-called "ropy saliva," which was found by him to be associated with the rapid white decay, which he attributed to nitric acid. This theory as such was accepted and put into practice by many, but it was no more successful than the other theory that all dentifrices should have a strong alkaline reaction, in order that the saliva might be kept in a normal physiological balance.

We predict that we shall have less caries when we can determine with sufficient exactness the character of food supplying a balanced saliva and are able to have our patients co-operate intelligently and faithfully; and at the same time we should not be in too great haste to discard the tooth brush and dentifrice. For the same reasons that we shall always use soap as a skin detergent we shall need our detergent dentifrices. Professor Kelly's paper makes a serious charge of professional neglect which is altogether too true to be ignored. The general public is not using the oral toilet means with any degree of scientific accuracy, and it is largely the dentists fault. If we have not done our duty any better than his paper seems to indicate, how can we expect that the more difficult problem of a prophylactic diet will be made known or enforced, even with the limited 10 percent. of our great country's population who come under dental influence. The subject is one of the greatest importance, and it is of tremendous significance. Are we ready, or even capable of grasping this problem, to give it to our patients in a practicable form, to say nothing of promulgating it for the masses who take no special thought for practical oral prophylaxis?

REORGANIZATION OF THE NEW YORK STATE SOCIETY. At its meeting held in May, the New York State Dental Society effected a reorganization which makes it possible for this society to join with other state societies in the reorganization of the National Dental Society. The New York Society has had a peculiar form of organization which it was supposed was so strongly entrenched, that it would be difficult, if not impracticable, to change so as to permit the carrying out of any scheme of reorganizing the National Society with its co-operation. It now seems certain that the other states with strong memberships which have already declared their intentions to unite for the National reorganization will make the scheme practicable. This will be the greatest step in advance the profession has ever taken and will certainly result in more unity of action and concentration of purpose in our professional affairs than has been practicable under former conditions. There will, of course, be the usual mistakes and disappointments incident to the launching of any large and new enterprise; but so much good thinking and planning, and with the precedents of the American Medical Association to help us, that we should make a fairly perfect and workable organization possible at once. We shall, however, need to be patient and charitable in our criticisms and allow time to adjust whatever may seem to be unsatisfactory. If every dentist will join his local or district society, and loyally support the national movement, we shall soon realize the benefits of a large and strong organization.

